# Muscle magnetic resonance imaging abnormality in neuroleptic malignant syndrome: a case report

**DOI:** 10.1186/s12883-022-02937-2

**Published:** 2022-10-29

**Authors:** Yuki Kakinuma, Ryota Amano, Atsushi Ishida, Ichizo Nishino, Katsumi Taki

**Affiliations:** 1 Department of Internal Medicine, Fujiyoshida Municipal Medical Center, Yamanashi, Japan; 2 Department of Neuromuscular Research, National Institute of Neuroscience, National Center of Neurology and Psychiatry, Tokyo, Japan

**Keywords:** Neuroleptic malignant syndrome, Schizophrenia, Antipsychotic drug, Muscle pathology, Magnetic resonance imaging

## Abstract

**Background:**

Neuroleptic malignant syndrome (NMS) is a rare and occasionally fatal undesirable reaction to dopamine antagonists, and its phenotype is diverse owing to causative drugs. Classically, elevation of serum creatine kinase is described in NMS. Some reports have described muscular pathological findings; however, muscle magnetic resonance imaging (MRI) has not been reported previously.

**Case presentation:**

A 63-year-old woman with a history of schizophrenia presented to our hospital with a high fever, excessive sweating, muscle weakness, and elevated serum creatine kinase levels. Muscle MRI revealed T2 high-intensity lesions in several muscles with gadolinium enhancement, and the pathology of the muscle biopsy showed a very mild presence of muscle fiber necrosis and regeneration with type 2c fibers without inflammation. Her symptoms resolved by treatment with levodopa/carbidopa, dantrolene. Finally, the patient was diagnosed with NMS.

**Conclusions:**

This is the first report of muscle MRI abnormalities in a patient with NMS. Muscle MRI abnormalities in NMS may be associated with non-inflammatory myopathic changes. The cause of creatine kinase elevation cannot be explained by abnormal strong muscle contraction nor inflammation.

## Background

Neuroleptic malignant syndrome (NMS) was first described after the introduction of antipsychotic drugs for the treatment of psychotic disorders in the late 1950s [1]. The incidence of NMS ranges from 0.01–0.02% among individuals treated with antipsychotic medications [2]. NMS is diagnosed by exclusion, and the symptom complex is broad. However, the following diagnostic criteria have been proposed: recent dopamine antagonist exposure, dopamine agonist withdrawal, hyperthermia, rigidity, mental status alteration, creatine kinase elevation, sympathetic nervous system lability, and tachycardia plus tachypnea [3]. One of the dominant mechanisms of NMS is the blockade of D2 dopamine receptors in the hypothalamus and brainstem regulatory systems, which leads to hyperthermia. In addition, D2 dopamine receptors in the striatum are involved in muscle rigidity, while those in the midbrain and cortical pathways are involved in changes in the mental state [4].

In NMS, cessation of antipsychotic medication and medical management are sufficient to resolve symptoms. Lorazepam, a benzodiazepine that activates the gamma-aminobutyric acid -A receptor and modulates its inhibition of neurotransmission, and levodopa, a precursor of dopamine that activates dopamine receptors, are known to be therapeutic agents for NMS [5, 6]. Bromocriptine and amantadine are dopamine agonists that inhibit antipsychotic dopamine antagonists [5]. Dantrolene, a specific drug that inhibits calcium ion release from the sarcoplasmic reticulum, is also effective in the treatment of muscle rigidity, hyperthermia, and serum creatine kinase (CK) elevation [6, 7]. Moreover, volume replacement should be aggressive, especially because most patients with NMS are dehydrated during the acute phase of the illness [2].

In addition to biopsy, magnetic resonance imaging (MRI) of muscle for the diagnosis and follow-up is also advocated in some myopathies [8]. In NMS, there have been some reports that muscle pathology shows edematous changes with or without necrotic changes, with no evidence of regeneration in autopsy studies [9, 10]. In addition, biopsy studies have revealed non-specific type 2 fiber atrophy and denervation atrophy [11, 12]. However, to the best of our knowledge, muscle MRI has not previously been performed for NMS. Here, we report a case of NMS that showed abnormal hyperintensity on muscle MRI and muscle pathology.

## Case presentation

A 63-year-old woman with a fever of > 38 °C, excessive sweating, bilateral muscle pain, and difficulty in moving was referred to our hospital in late spring. She had a 30-year history of schizophrenia and was treated with antipsychotic medications such as diazepam, ethyl loflazepate, biperiden, asenapine, and risperidone. Although she regularly took the prescribed drugs, her hallucinations worsened. Her family doctor had started a monthly intramuscular injection of haloperidol into the gluteus maximus muscle 5 months before she visited our hospital, and she had received the last injection 12 days before visiting our hospital. She had a low fever in the 37 °C range on the night before hospitalization. Her fever increased to > 38 °C in the morning of the hospitalization day, and she requested an ambulance at 7am. It was rainy the day before hospitalization, and the highest external temperature was 22 °C. She cared for her aged mother and lived independently at home prior to hospitalization. She had no history of excessive exercise, convulsions, or the use of statins or fibrate-based medicines. Moreover, the patient had no family history of muscular disease. The time from fever to ambulance transport was no longer than 12 h, and she could turn over in her bed before hospitalization. On admission, her vital signs were as follows: body temperature of 38.0 °C; heart rate of 78 beats/min; respiration rate of 20 breaths/min; and blood pressure of 107/55 mmHg. Her oxygen saturation level was 97% in ambient air.

**Fig. 1:**
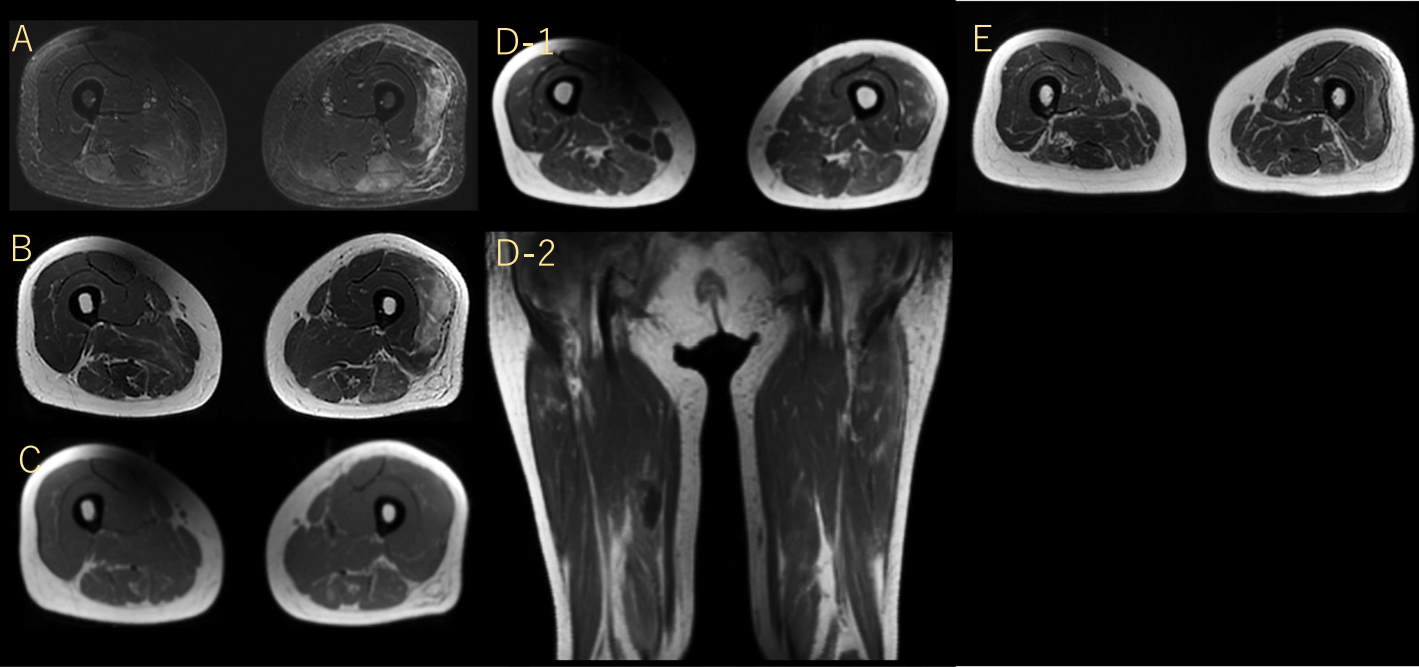
Femoral MRI images before **A**–**D** and after **E** muscle biopsy. STIR image (**A**). T2-weighted image (**B**). T1-weighted image (**C**). Gadolinium-enhanced image (axial; D-1, coronal; D-2, respectively). Follow up MRI on day 33 (T2-weighted image) (**E**). MRI: magnetic resonance imaging, STIR: short T1 inversion recovery

**Fig. 2:**
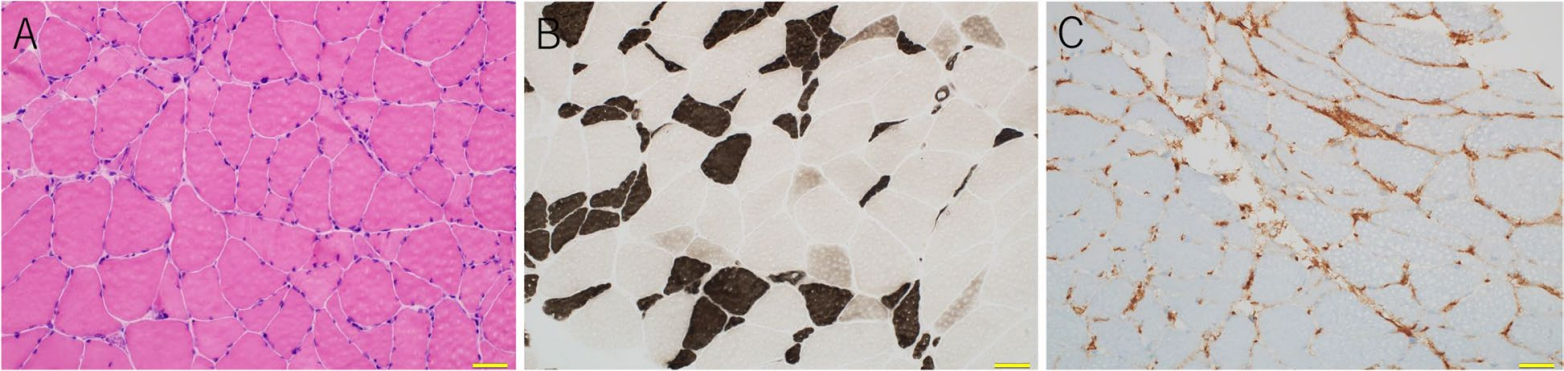
Pathological findings. Hematoxylin–eosin staining (**A**). Adenosine triphosphatase staining (**B**). HLA-ABC immunohistochemical staining (**C**). Images were taken using an Olympus BX53 microscope equipped with 20 × objective lens and DP74 camera using Cell Sense software with a resolution of 0.46 µm/pixel. Any adjustments were applied to the entire image. Threshold manipulation, expansion or contraction of signal ranges, and altering of high signals were not performed. The scale bar length is 50 µm. HLA-ABC: Human leukocyte antigen-ABC

Physical examination revealed muscle pain, diffuse muscular weakness (manual muscle test score, 4/4), and slight systemic tremor; no disturbance in consciousness or muscular rigidity was observed. Her slight systemic tremor was consistent with drug-induced Parkinsonism. She reported relatively strong muscle pain that could disturb her movement in the posterior region of the neck, bilateral shoulders, ventral and dorsal upper and lower arms, and ventral and dorsal thighs. Essentially, muscle pain was experienced in both the dorsal and ventral muscles. She required assistance from medical workers whether she was in a sitting or standing position. Laboratory examinations revealed that serum CK levels were markedly elevated (12,426 U/L; reference values 0–170 U/L). Qualitative analysis of urine using a test strip revealed strongly positive hemoglobin, but no erythrocytes were found in the sediment. These observations strongly suggested myoglobinuria. The inflammatory responses were mildly elevated (white blood cell (WBC) count of 9830 /μL and C-reactive protein (CRP) of 0.96 mg/dL). Liver, renal, and thyroid functions were within normal ranges, except for mild dehydration (blood urea nitrogen of 34.7 mg/dL and creatinine of 0.67 mg/dL). The patient’s serum tested negative for antinuclear antibodies, anti-aminoacyl transfer ribonucleic acid synthetase antibodies, antimitochondrial M2 antibodies, and human T-cell leukemia type 1 antibodies. Contrast-enhanced computed tomography (CT) from the chest to the pelvis showed no obvious inflammatory sources or tumorous lesions, and head CT showed no obvious abnormalities in the brain. Transthoracic echocardiography revealed no diastolic left ventricular dysfunction or left ventricular wall with a well-preserved ejection fraction (70%). Her highest fever was 39.1 °C on day 3. On day 5, femoral MRI of short T1 inversion recovery (STIR) and T2-weighted images also revealed heterogeneous high-intensity lesions in the bilateral biceps femoris, semimembranosus, left vastus lateralis, right adductor magnus muscles, and subcutaneous tissues (Fig. 1A, B). T1-weighted imaging showed isointense lesions in the same muscle (Fig. 1C). Gadolinium-enhanced T1-weighted images showed dot-like and/or linear streaky enhancement on the left vastus lateralis, biceps femoris, and semimembranosus muscles, and rim enhancement on the right adductor magnus muscle (Fig. 1D-1, D-2). Needle electromyography was not performed. Based on the results of laboratory examinations and a history of taking antipsychotic medications, we suspected NMS. However, we also considered that the lack of disturbance of consciousness and rigidity was atypical of NMS at that time. The muscle MRI abnormalities and muscle pain were consistent with inflammatory myositis, and it was necessary to consider the possibility of inflammatory myositis. In addition, her hallucinations relapsed because of the discontinuation of antipsychotic drugs. To determine the appropriate treatment, we needed to check the muscle pathology. Therefore, we performed a biopsy of the left vastus lateralis muscle.

On muscle pathology, no necrotic and regenerating fibers, as well as mononuclear cell infiltration, were seen (Fig. 2A). Nevertheless, scattered type 2C fibers were observed in addition to moderate type 2 fiber atrophy on myosin adenosine triphosphatase (ATPase), suggesting the presence of minimal myofiber necrotic and regenerating process (Fig. 2B). Human leukocyte antigen-ABC (HLA-ABC) was only minimally expressed in some muscle fibers (Fig. 2C). Although not completely normal, these findings were not suggestive of any particular neuromuscular disorders including inflammatory myopathies such as dermatomyositis and immune-mediated necrotizing myopathy.

We discontinued all antipsychotic medications and initiated levodopa/carbidopa and dantrolene therapy. Her autonomic symptoms (fever and excessive sweating) lasted for at least 6 days but were gradually relieved. In particular, the patient clearly commenced recovery soon after the initiation of levodopa/carbidopa. Her CK levels declined to 104 U/L on day 21, and her muscle pain was relieved. The follow-up MRI on day 33 showed diffuse muscular atrophy compared to the previous MRI (Fig. 1E). Because her auditory hallucinations persisted, we initiated treatment with quetiapine fumarate. During her hospitalization, she underwent as much rehabilitation as possible. The patient was able to walk alone and was discharged on day 34.

## Discussion and conclusions

NMS is generally characterized by high fever, muscle rigidity, and autonomic symptoms. However, in atypical cases, these typical abnormal presentations are often absent or develop slowly [13, 14]. Moreover, rigidity is not required for diagnosis. Trollor et al. reported that the rate of rigidity was 33.7% (48.8% in 1^st^ generation antipsychotic and 29.7% in 2^nd^ generation antipsychotic drugs) [15]. In the present case, the lack of consciousness disturbance might be explained by the regular use of benzodiazepines (diazepam and ethyl loflazepate). Although evident muscular rigidity was not observed, the clinical diagnosis of NMS was definite because no other diseases could explain her clinical symptoms (high fever and excessive sweating), laboratory data (hyperCKmia, mildly elevated CRP, and WBC), muscle pathology, and favorable response to levodopa/carbidopa-based medication therapy. Antipsychotic medications should be discontinued to provide a better treatment for NMS. However, in some cases, antipsychotic medications are essential for controlling mental manifestations. The relapse rate is estimated to be 17–30% when antipsychotic medications are restarted after NMS has disappeared [16]. Ananth et al. reported that NMS did not recur if the same drug was re-administered at least 4 weeks after recovery from the initial attack [17]. Therefore, it is advisable to allow as much time as possible, even if the same antipsychotic must be re-administered. Re-administration of antipsychotics is often necessary, but should be attempted with a different agent after a period of no medication and titrated slowly with close monitoring [18]. In this case, we decided to prescribe a different medication because auditory hallucinations due to schizophrenia reappeared strongly after antipsychotic medications were discontinued.

In NMS, the muscle pathology is reported to show nonspecific type 2 fiber atrophy and denervation atrophy [11, 12]. Type 2 fiber atrophy occurs in various conditions, such as sarcopenia, aging, steroid myopathy, and central nervous system disorders, and is not associated with specific diseases [19]. In the present case, the muscle pathology of biopsy suggested moderate type 2 fiber atrophy, and a very mild process of muscle fiber necrosis and regeneration with type 2c fibers, which indicate regenerating or fetal muscle fibers between denervation and reinnervation processes in the background [20]. However, fiber-type grouping, which suggests a reinnervation process in regenerating muscles, was not apparent. Therefore, the presence of type 2c fibers might indicate rhabdomyolysis or a very acute phase of denervation.

None of the previous reports have described myopathic changes in NMS as “inflammation”. In the present case, HLA-ABC, which indicates inflammatory changes, was rarely expressed. If the muscle pathology of NMS is mainly related to inflammatory changes, HLA-ABC expression should be stronger. Therefore, muscle MRI abnormalities in NMS may be associated with noninflammatory myopathic changes.

On muscle MRI, the presence of high T2/STIR signals generally indicates edematous change, inflammation, or acute denervation [8, 20, 21]. Lu et al. reported two distinct imaging types of rhabdomyolysis. Type 1 rhabdomyolysis is caused by overexercise, and the affected muscles show homogeneous signal changes on T2 and STIR images, and homogenous enhancement by gadolinium. In contrast, type 2 rhabdomyolysis is caused by direct trauma, vessel occlusion, and altered metabolism, and the affected muscles show heterogeneous signal changes on T2 and STIR images. Rim-enhanced or dot-like and/or linear streaky enhancement of gadolinium enhancement has been reported [22]. Retrospectively, the MRI findings of our patient did not contradict those of type 2 rhabdomyolysis. Therefore, we suspected that the mechanisms of hyperCKmia might be associated with the altered metabolism in muscle cells.

Although the mechanisms of muscle injury are not completely clear, one of the possible mechanisms seems to be related to the increase in free intracellular calcium due to damage to the muscle sarcolemma and failure of the energy supply within the muscle cell. Despite the causes of rhabdomyolysis are numerous, the final pathogenic pathway is commonly dependent on the increase of free ionized calcium in the cytoplasm. The increased calcium initiates a chain of downstream reactions (activation of calcium-dependent proteases and phospholipases, mitochondrial dysfunction) that eventually lead to the destruction of the muscle cell [23].

To the best of our knowledge, muscle MRI has not previously been performed for NMS. As the pathological findings were negative for inflammation, we considered that the abnormally high T2/STIR intensities on MRI did not reflect inflammation. Moreover, because this patient showed hyperCKmia without muscular rigidity, the mechanisms of hyperCKmia could not be caused by abnormally strong muscle contraction. This is the first report describing the muscle MRI abnormality of NMS. When a patient taking antipsychotic medication shows high T2/STIR intensities on muscle MRI, physicians should consider the possibility of NMS.

## Data Availability

All data related to this case report are contained within the manuscript.

## Abbreviations

CK: Creatine kinase
CRP: C-reactive protein
CT: Computed tomography
HLA-ABC: Human leukocyte antigen-ABC
MRI: Magnetic resonance imaging
NMS: Neuroleptic malignant syndrome
STIR: Short T1 inversion recovery
WBC: White blood cell
